# The cancer ratio plus in the differential diagnosis of pleural effusions: a scoping review of current evidence

**DOI:** 10.11613/BM.2026.010502

**Published:** 2025-12-15

**Authors:** Yasmine Bendimrad, Lamia Mellah, Malak Snoussi, Jalila El Bakkouri

**Affiliations:** 1 Mohammed VI Faculty of Pharmacy, Mohammed VI University of Sciences and Health, Casablanca, Morocco; 2 Laboratory Medicine, Cheikh Khalifa International University Hospital, Mohammed VI University of Sciences and Health, Casablanca, Morocco; 3 Mohammed VI Faculty of Medicine, Mohammed VI University of Sciences and Health, Casablanca, Morocco; 4 Immunopathology-Immunotherapy-Immunomonitoring Laboratory, Mohammed VI University of Sciences and Health, Casablanca, Morocco

**Keywords:** cancer ratio plus, malignant pleural effusion, tuberculous pleural effusion, differential diagnosis

## Abstract

**Introduction:**

Differentiating between malignant pleural effusion (MPE) and tuberculous pleural effusion (TPE) remains challenging in clinical practice. The cancer ratio plus (CR+), a potential diagnostic tool calculated as serum lactate dehydrogenase/(pleural adenosine deaminase x pleural lymphocyte percentage) has emerged to address this diagnostic challenge. This scoping review maps the available evidence on its diagnostic performance.

**Materials and methods:**

We conducted a systematic search of PubMed, Scopus, and Web of Science databases from inception to April 2025. Eligible studies assessed the accuracy of CR+ in distinguishing MPE from TPE. Data on study design, cut-off values, sensitivity, specificity, area under the curve (AUC), and likelihood ratios were extracted and synthesized narratively.

**Results:**

Six studies comprising 881 patients were included. Reported cut-off values varied widely (5.7 - 41.0), as did sensitivity (74.3 - 97.6%) and specificity (36.6 - 94.1%). Most studies, however, reported good discriminatory power with AUC values generally above 0.80. The highest diagnostic accuracy was observed in one study, which reported a sensitivity of 97.6%, a specificity of 94.1%, and an AUC of 0.86. Differences in cut-off thresholds, study populations, local tuberculosis epidemiology, and laboratory methodology (particularly lymphocyte quantification) likely contributed to this heterogeneity.

**Conclusions:**

The CR+ appears promising as a non-invasive tool using routine parameters for differentiating MPE from TPE, but diagnostic performance varies across settings. The heterogeneity in optimal cut-off values highlights the need for local validation before clinical adoption. Future research should standardize methodology and assess its impact on decision-making and patient outcomes.

## Graphical abstract

**Figure:**
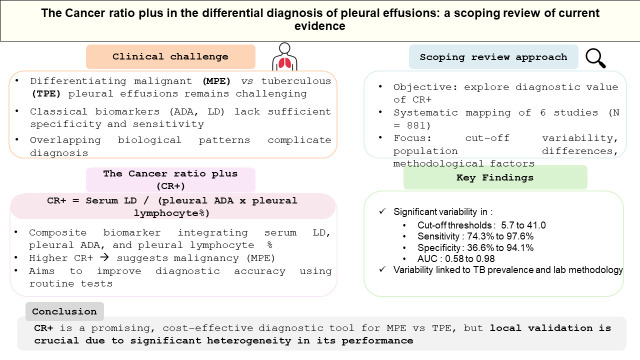

## Highlights

• The calculated cancer ratio plus (CR+) appears promising for differentiating malignant from tuberculous pleural effusions

• This review maps available evidence and identifies gaps in clinical practice

• The composite nature of CR+ (using three routine parameters) offers a potential advantage over single conventional markers

Findings support future research to validate CR+

## Introduction

Pleural effusion is a common clinical condition characterized by the abnormal accumulation of fluid within the pleural space (*1*). It represents a significant diagnostic challenge in clinical practice globally, particularly in regions with high tuberculosis (TB) prevalence (*2*). Among its diverse etiologies, malignant pleural effusion (MPE) and tuberculous pleural effusion (TPE) are of particular clinical importance due to their vastly different management strategies and prognostic implications (*3*, *4*). Accurate and timely differentiation between these two conditions is particularly challenging because both typically present as lymphocyte-predominant exudative effusions with similar gross appearance and overlapping biochemical profiles on initial laboratory analysis, making clinical distinction difficult without additional specialized testing (*5*). Misdiagnosis can lead to delays in appropriate cancer treatment for MPE or unnecessary prolonged anti-TB therapy for TPE, both of which can negatively impact patient outcomes (*6*).

The diagnosis of pleural effusion typically involves thoracentesis followed by pleural fluid analysis, including cytological examination and microbiological tests (*7*, *8*). While cytology is crucial for identifying malignant cells, its sensitivity is limited, particularly in effusions with low cellularity or specific cancer types (*9*). Similarly, the prolonged turnaround time for microbiological cultures, essential for confirming tuberculous etiology, can lead to clinically significant diagnostic delays (*10*). Moreover, the diagnostic challenge for TPE is compounded because tuberculous pleuritis typically involves a paucibacillary process where only a scant number of bacilli are present in the pleural fluid, resulting in the low sensitivity of standard microbiological tests and frequent negative culture results (*11*). These inherent limitations underscore the urgent need for rapid and accurate complementary diagnostic tools.

Classical biomarkers in pleural fluid have been commonly used to aid in the differentiation of MPE and TPE (*12*). Pleural fluid adenosine deaminase (ADA) activities are typically higher in TPE, while lactate dehydrogenase (LD) tends to be higher in MPE (*13*, *14*). Adenosine deaminase is an intracellular enzyme involved in purine metabolism and is released predominantly by activated T lymphocytes and macrophages during cellular immune responses (*15*, *16*). Higher ADA in TPE reflects the intense T-cell-mediated immune activation within the pleural space (*17*). However, higher ADA is not specific, as MPE can also exhibit moderately higher ADA due to increased lymphocyte turnover in the tumor microenvironment and by the tumor cells themselves, thus decreasing its specificity for TPE (*18*). Lactate dehydrogenase, on the other hand, is a cytoplasmic enzyme present in virtually all tissues and released into circulation following cellular injury or necrosis (*19*). Higher serum LD activities are commonly observed in malignancy, reflecting both the high metabolic activity of tumor cells and the extensive cellular turnover associated with cancer progression (*20*). Conversely, in tuberculous pleurisy, serum LD is generally lower, as the process is dominated by localized immune activation rather than widespread tissue destruction. Other biochemical parameters, such as protein concentrations, glucose, and differential cell counts, are also frequently employed to enhance diagnostic accuracy (*14*). However, despite their clinical usefulness, all these conventional biomarkers suffer from limited sensitivity and specificity.

To overcome these diagnostic limitations and improve diagnostic accuracy, the cancer ratio - defined as the ratio of serum LD to pleural fluid ADA - was introduced as a composite diagnostic index (*21*). Studies suggest that a high cancer ratio may effectively indicate MPE, although diagnostic accuracy varies in the literature. Building upon this approach, the Cancer ratio plus (CR+) was developed to further enhance differentiation. This modified index integrates three routinely measured laboratory parameters - serum LD, pleural ADA, and pleural lymphocyte percentage - to improve discrimination between malignant and tuberculous effusions. It is calculated according to the following formula: CR+ = serum LD/(pleural ADA x pleural lymphocyte percentage) (*22*). The rationale behind this modification is that pleural lymphocyte percentages are usually lower in malignant effusions compared to tuberculous effusions, thus potentially improving diagnostic differentiation (*22*).

However, despite growing interest, the available evidence remains limited and inconsistent across studies. To date, no comprehensive synthesis has specifically focused on evaluating the diagnostic value of the CR+. The objective of this scoping review is therefore to systematically map the existing evidence on the diagnostic performance of the CR+ in differentiating malignant from tuberculous pleural effusions. Specifically, we aim to summarize reported cut-off values and diagnostic accuracy metrics, and to highlight gaps in current literature that warrant further research.

## Materials and methods

### Protocol and registration

This scoping review was conducted following the PRISMA-ScR (Preferred Reporting Items for Systematic Reviews and Meta-Analyses extension for Scoping Reviews) guidelines, a framework designed to ensure transparent and comprehensive reporting of scoping reviews (*23*). The protocol was prospectively registered on the Open Science Framework (https://doi.org/10.17605/OSF.IO/VBG8Y) on April 16. 2025.

### Search strategy

A comprehensive literature search was performed across three electronic databases: PubMed, Scopus, and Web of Science, from inception to April 2025. The search strategy combined terms related to pleural effusions, malignancy, tuberculosis, and the CR+. Specifically, keywords included combinations of “pleural effusion,” “malignant pleural effusion,” “tuberculous pleural effusion,” “Cancer Ratio Plus,” “serum LDH,” “pleural ADA,” “lymphocyte percentage,” and diagnostic accuracy terms such as “diagnosis,” “sensitivity,” and “specificity.” Additionally, a manual search of reference lists was performed, which identified one additional article published in 2025.

### Eligibility criteria

Studies were eligible for inclusion if they met the following criteria:

Reported on the diagnostic performance of the CR+ for differentiating between MPE and TPE.
Provided at least one diagnostic accuracy metric. The following diagnostic accuracy metrics were considered: sensitivity, specificity, and area under the receiver operating characteristic curve (AUC). Positive likelihood ratio (PLR) and negative likelihood ratio (NLR) were also included when available.
Were original research articles published in English.

Case reports, reviews, editorials, and studies focusing solely on cancer ratio (without the “Plus” modification) were excluded, as they did not provide original data on the diagnostic performance of the specific diagnostic tool of interest.

### Selection process

**Figure 1:**
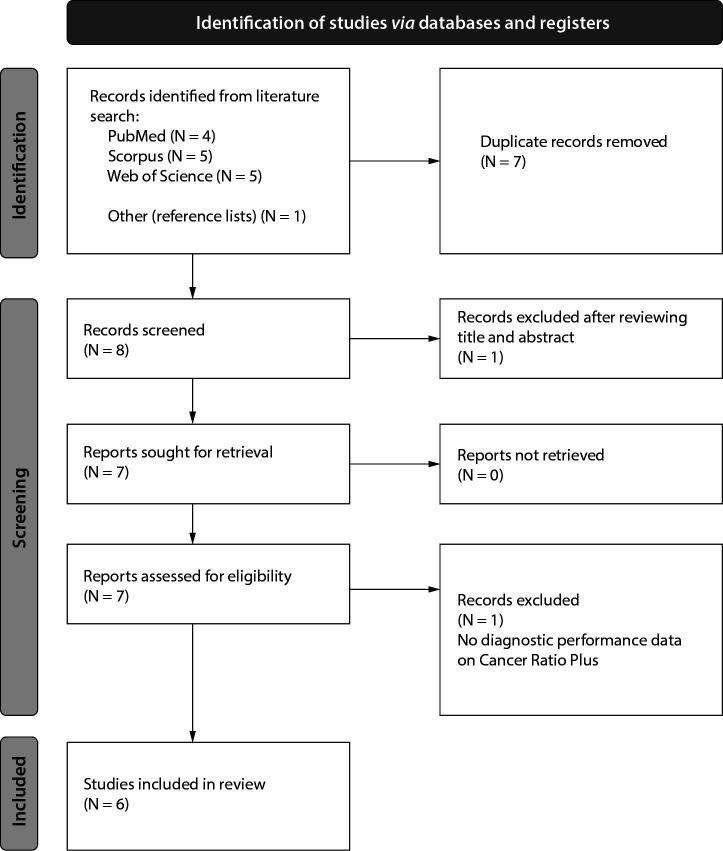
PRISMA 2020 flow diagram illustrating the study selection process for the scoping review.

Duplicate records were identified and removed using Microsoft Excel. Titles and abstracts were screened independently by a single reviewer, followed by full-text review. Any uncertainties were resolved through discussion with a senior supervisor. The study selection process is illustrated in the PRISMA flowchart (Figure 1). During the screening phase, we noted a limited number of published articles specifically evaluating the CR+, highlighting the novelty of this diagnostic tool and the relevance of conducting this scoping review.

### Data extraction and charting

A standardized data extraction form was used to collect the following data: first author, year of publication, and country, study design, characteristics of the study population and comparator groups (MPE *vs.* TPE cases), reported cut-off value for the CR+, diagnostic performance metrics: sensitivity, specificity, AUC, PLR, and NLR values (if available) and whether the individual components (serum LD, pleural ADA, lymphocyte percentage) were reported separately.

### Data synthesis

The extracted data were synthesized narratively and presented in tables. No meta-analysis was performed due to the anticipated heterogeneity across study designs, populations, and diagnostic thresholds. This approach allows for a comprehensive description of the available evidence while acknowledging the limitations of combining the data statistically. Key findings, including reported cut-off values and diagnostic accuracy metrics, were summarized to provide an overview of the current evidence regarding the CR+.

## Results

### Study selection and characteristics

A total of six studies met the inclusion criteria and were included in this scoping review. This relatively small number of studies highlights the limited current evidence on the CR+. The selection process is detailed in the PRISMA-ScR flow diagram (Figure 1). The included studies were published between 2016 and 2025.

Most studies employed a prospective observational design, which generally provides stronger evidence than retrospective studies, while two were retrospective. The studies were conducted across various geographic regions, including Asia (Singapore, China, India), Africa (Egypt), and Europe (Turkey), indicating a reasonable geographic distribution. Sample sizes varied considerably across studies, ranging from 78 to 220 participants (estimated median sample size: 132). The comparator groups also differed, with some studies comparing only MPE *vs.* TPE, while others included additional diagnostic categories such as parapneumonic effusions, which may contribute to heterogeneity in the results.

### Data extraction and diagnostic performance

The key findings related to cut-off thresholds and diagnostic performance are summarized below and presented in detail in Table 1. Cut-off thresholds for the CR+ showed a substantial variation across included studies, ranging from 5.65 to 41.00. Sensitivity values varied considerably, ranging from 74.3% to 97.6%, while specificity also showed marked variation, ranging from 36.6% to 94.1%. The area under the receiver operating characteristic curve, a measure of the test’s overall discriminatory ability, was reported in all studies, with values ranging from 0.58 to 0.98, indicating generally good to excellent discrimination. Positive likelihood ratio and NLR were also available for most studies, although some did not report these metrics explicitly.

**Table 1:** Diagnostic performance of cancer ratio plus in included studies

**Study**	**Country**	**Study design**	**N**	**Comparator group**	**Cut-off (CR+)**	**Sensitivity (%)**	**Specificity (%)**	**AUC**	**PLR**	**NLR**
Verma *et al.*, 2016 (*22*)	Singapore	Prospective	118	MPE *vs.* TPE	> 30	97.6	94.1	0.86	41	0.06
Hussein and Elhefnawy, 2020 (*29*)	Egypt	Prospective	78	MPE *vs.* TPE *vs* others	≥ 41	93.6	91.7	0.98	NR	NR
Ren and Xu, 2021 (*30*)	China	Retrospective	219	MPE *vs.* TPE	> 22.6 (for > 50 years)	86.8	84.6	0.84	5.64	0.16
Zhou *et al.*, 2022 (*31*)	China	Prospective	220	MPE *vs.* TPE	≤ 34.74*	81.5	80.0	0.87	4.06	0.23
Gayaf *et al.*, 2021 (*32*)	Turkey	Retrospective	146	MPE *vs.* TPE *vs.* PPE	> 36.88	74.3	88.9	0.69	6.68	0.29
Parida *et al.*, 2025 (*33*)	India	Prospective	100	MPE *vs.* TPE *vs.* PPE	> 5.65	89.7	36.6	0.58	1.41	0.28
*For the Zhou *et al.* (2022) study, the CR+ was evaluated as a negative predictive factor for TPE. Thus, a cut-off of ≤ 34.74 indicates TPE, implying that values > 34.74 suggest MPE. N - sample size. MPE - malignant pleural effusion. TPE - tuberculous pleural effusion. PPE - parapneumonic effusion. CR+ - cancer ratio plus. PLR - positive likelihood ratio. NLR - negative likelihood ratio. AUC -area under the curve. NR - not reported.										

## Discussion

This scoping review mapped current evidence on the diagnostic utility of the CR+ for distinguishing MPE from TPE. Across six studies encompassing 881 patients from diverse geographic settings, the CR+ consistently demonstrated promising diagnostic performance. Most studies reported AUC values above 0.80, indicating good to excellent discriminatory power. However, considerable heterogeneity was observed in cut-off thresholds, which ranged from 5.7 to 41.0, as well as in sensitivity (74.3-97.6%) and specificity (36.6-94.1%).

This variability likely reflects differences in study populations, laboratory protocols, clinical settings, and disease prevalence, thereby limiting the generalizability of a universal threshold. Nevertheless, the consistent trend toward improved diagnostic discrimination compared to conventional markers highlights the CR+ as a promising tool warranting further validation.

The CR+ builds upon two widely accessible biomarkers - serum LD and pleural ADA - by incorporating pleural lymphocyte percentage, a marker typically elevated in TPE. This composite approach captures multiple pathophysiological dimensions and appears to enhance diagnostic accuracy. In several studies, the CR+ outperformed both ADA alone and the original Cancer Ratio (serum LD/pleural ADA). Notably, Verma *et al.* and Zhou *et al.* found that CR+ provided superior performance when integrated with traditional markers.

This multi-parameter model may reduce false positives in settings where ADA activities are higher in non-tuberculous conditions or where LD alone lacks specificity (*24*). The addition of lymphocyte percentage may also be particularly useful in borderline cases, increasing clinician confidence in differential diagnosis.

Several factors may explain the substantial heterogeneity observed across studies. Geographic differences play a significant role, as the prevalence of TB and malignancies varies widely between countries and influences the pre-test probability of each condition (*25*). Patient selection criteria also differed between studies: some included only lymphocytic exudates, while others assessed a broader spectrum of pleural effusions, including parapneumonic or indeterminate causes.

A notable source of heterogeneity arises from the study by Parida *et al.* (2025), which reported the lowest specificity (36.6%) and a poor discriminatory performance (AUC = 0.58), which the authors themselves reported as non-significant (P = 0.171). This contrasts with all other studies, which demonstrated AUC values ranging from 0.69 to 0.98. This outlier pattern can be explained by both statistical and epidemiological factors.

From a statistical perspective, the authors used an exceptionally low cut-off threshold (5.7) compared with other studies (ranging from 22.6 to 41.0). Lower thresholds classify more patients as test-positive, which mathematically increases sensitivity (detecting nearly all true positives, 89.7%) but inevitably lowers specificity, because more false positives are inevitably captured - specifically, TPE or parapneumonic pleural effusion misclassified as malignant. This sensitivity-specificity trade-off is a well-established principle in diagnostic testing and fully accounts for the pattern observed in this study (*26*).

From an epidemiological standpoint, the research was conducted in Odisha, India - a region within a country with one of the world’s highest TB burdens (*27*). In this context, almost half of the cohort (48%) presented TPE, while malignant effusions represented only 29%. Such a high TB prevalence fundamentally shifts the pre-test probability and can reduce the discriminative capacity of the CR+. Additionally, regional differences in TB strains, patient characteristics (including comorbidity profiles, nutritional status, and age distributions), local disease spectrum, cancer types, and possibly pleural fluid handling or laboratory measurement protocols may all have contributed to the observed discrepancies. The inclusion of parapneumonic pleural effusions (23% of cases) as a third diagnostic category may have further complicated the diagnostic landscape.

Analytical and methodological variability represents another major source of heterogeneity. While ADA and LD assays are generally well-standardized, pleural fluid cell differential reporting remains far more variable across laboratories. In particular, the quantification of pleural fluid lymphocytes, a key denominator component of the CR+ formula, lacks standardization. While all included studies reported "lymphocyte percentage", none provided sufficient detail regarding differential cell counting methodology (manual *vs*. automated) or specific morphological criteria used to distinguish lymphocytes from monocytes or reactive macrophages. These methodological differences in lymphocyte identification and counting could introduce systematic variation in CR+ values across laboratories and contribute to the observed heterogeneity in optimal cut-off thresholds.

Age demographics are another important variable. For example, Ren *et al.* highlighted that age-specific thresholds may be necessary, especially since the prevalence of malignancy increases with advancing age (*28*).

Overall, these findings suggest that local epidemiology significantly influences the diagnostic relevance of CR+, with substantially reduced or negligible utility possible in certain high TB-burden settings. Rather than undermining previous studies, the discordant result from Parida *et al.* reinforces their validity by highlighting the crucial importance of interpreting CR+ within its specific clinical, methodological, and epidemiological context. These factors collectively underscore that no universal CR+ threshold can be applied across all clinical contexts, and that rigorous local validation aligned with population characteristics and clinical priorities is essential for successful implementation in routine practice. Future systematic reviews and meta-analyses should therefore stratify results by TB prevalence, geographic region, and population characteristics rather than pooling heterogeneous data.

Despite its limitations, the CR+ offers several pragmatic advantages. It is based entirely on laboratory parameters that are already measured as part of standard pleural fluid analysis, requiring no specialized equipment or additional cost. This makes it particularly attractive in resource-limited settings, where advanced diagnostic techniques such as thoracoscopy may be inaccessible. The test also provides rapid results, potentially expediting decision-making and avoiding unnecessary treatment delays.

However, the lack of standardized cut-off values remains a key obstacle to clinical implementation. Applying thresholds derived from dissimilar populations or methods could lead to misclassification and inappropriate clinical decisions. Therefore, the CR+ should always be interpreted within its local epidemiological and laboratory context. Furthermore, this diagnostic tool should not be used in isolation, but rather as part of an integrated diagnostic approach that includes clinical assessment, radiological findings, and conventional microbiological or cytological testing.

Its optimal use may lie in serving as a triage tool or adjunct within diagnostic algorithms, particularly in patients with indeterminate cytology or equivocal ADA results. Incorporating the CR+ into clinical pathways may ultimately reduce the need for invasive procedures and facilitate earlier initiation of appropriate therapy.

This review is, to our knowledge, the first synthesis focused exclusively on the CR+. By aggregating results from diverse clinical and geographic contexts, it offers a comprehensive view of current evidence and identifies key knowledge gaps.

Nevertheless, several limitations must be acknowledged. First, the relatively small number of included studies (N = 6) limits the generalizability of our findings and precludes robust statistical synthesis. Second, our restriction to English-language publications may have led to the exclusion of relevant data from high TB burden regions such as parts of Eastern Europe, Central Asia, and the former Soviet Union countries, where valuable experience may not be published in English.

Third, stratified analyses by TB prevalence or regional epidemiology were not feasible. Notably, Parida *et al.* reported poor discriminatory ability in high TB-burden India (AUC 0.58), contrasting sharply with excellent performance elsewhere (AUC 0.69-0.98). This suggests CR+ may have limited utility in certain high-burden contexts. Inconsistent reporting across studies prevented further subgroup analyses.

Fourth, no formal assessment of study quality was conducted, and the heterogeneity in design and reporting prevented a meta-analytic approach. Importantly, most studies did not assess the real-world clinical impact of CR+, such as changes in diagnostic yield, decision-making, or patient outcomes. These limitations should inform the design of future research efforts.

To fully realize the potential of the CR+, future research should prioritize methodological standardization and context-specific validation. Specifically, studies should aim to harmonize ADA and LD assay protocols and establish robust, population-specific cut-off values tailored to local epidemiology and clinical priorities. Large-scale, prospective, multicenter trials are needed that deliberately recruit populations from both high and low TB-burden settings, with stratified analyses by TB prevalence, age groups, and cancer types. Additionally, efforts should be made to standardize lymphocyte counting methodology across laboratories to improve the reproducibility and comparability of CR+ values.

Equally important is the need to evaluate how CR+ influences clinical outcomes, including diagnostic efficiency, time to appropriate therapy, treatment decisions, and patient prognosis. The integration of CR+ into comprehensive diagnostic algorithms - alongside clinical, imaging, and potentially molecular biomarkers - may further refine its utility and contribute to a more personalized approach to pleural effusion management.

In conclusion, the CR+ emerges as a promising, accessible, and cost-effective diagnostic tool for distinguishing MPE from TPE. It offers diagnostic value beyond conventional markers, particularly in settings with limited diagnostic resources. However, its clinical use should be guided by locally validated thresholds and integrated into broader diagnostic frameworks. Rigorous prospective research is required to address existing limitations and to optimize its implementation in clinical practice.

## Data Availability

All data generated and analyzed in the presented study are included in this published article.
